# Gene Expression in Single Cells Isolated from the CWR-R1 Prostate Cancer Cell Line and Human Prostate Tissue Based on the Side Population Phenotype

**DOI:** 10.4172/2168-9431.1000150

**Published:** 2016-09-12

**Authors:** Kalyan J Gangavarapu, Austin Miller, Wendy J Huss

**Affiliations:** 1 Department of Pharmacology and Therapeutics, Roswell Park Cancer Institute, Buffalo, NY-14263, USA; 2 Department of Biostatistics, Roswell Park Cancer Institute, Buffalo, NY-14263, USA; 3 Department of Urologic Oncology, Roswell Park Cancer Institute, Buffalo, NY-14263, USA

**Keywords:** Single cells, Human prostate clinical specimens, Side population assay, ABCG2, Aldehyde dehydrogenase, Androgen receptor

## Abstract

Defining biological signals at the single cell level can identify cancer initiating driver mutations. Techniques to isolate single cells such as microfluidics sorting and magnetic capturing systems have limitations such as: high cost, labor intense, and the requirement of a large number of cells. Therefore, the goal of our current study is to identify a cost and labor effective, reliable, and reproducible technique that allows single cell isolation for analysis to promote regular laboratory use, including standard reverse transcription PCR (RT-PCR).

In the current study, we utilized single prostate cells isolated from the CWR-R1 prostate cancer cell line and human prostate clinical specimens, based on the ATP binding cassette (ABC) transporter efflux of dye cycle violet (DCV), side population assay. Expression of four genes: ABCG2; Aldehyde dehydrogenase1A1 (ALDH1A1); androgen receptor (AR); and embryonic stem cell marker, Oct-4, were determined.

Results from the current study in the CWR-R1 cell line showed ABCG2 and ALDH1A1 gene expression in 67% of single side population cells and in 17% or 100% of non-side population cells respectively. Studies using single cells isolated from clinical specimens showed that the Oct-4 gene is detected in only 22% of single side population cells and in 78% of single non-side population cells. Whereas, AR gene expression is in 100% single side population and non-side population cells isolated from the same human prostate clinical specimen.

These studies show that performing RT-PCR on single cells isolated by FACS can be successfully conducted to determine gene expression in single cells from cell lines and enzymatically digested tissue. While these studies provide a simple yes/no expression readout, the more sensitive quantitative RT-PCR would be able to provide even more information if necessary.

## Introduction

Single cell approaches are becoming increasingly useful because intra- and inter-tumoral genomic heterogeneity is difficult to analyze when studied in bulk cell populations [1, 2]. Studying intra- and inter-tumoral heterogeneity identifies targeted therapies and thus can improve clinical outcome. In a recent study conducted by Ennen et al. the authors studied single cell-specific gene expression profiles and were able to identify heterogeneous gene expression and specific genetic signatures that define clonal subpopulations based on their invasive and proliferative properties in melanoma cell lines [3]. Single cell approaches are also helpful in identifying rare sub clones that are involved in the emergence of resistance to chemotherapy and/or other types of therapy [1]. Identifying genetic and molecular characteristics of single cells would elucidate: (i) the hierarchical organization of different tissue types [4–6]; (ii) identification of markers for different cellular lineages in a given tissue type; (iii) the complex biological phenomena such as the relationship between cellular organization and genome function [7]; and (iv) define subpopulations in a cancer based upon their invasive, proliferative, and drug resistance properties [3].

Recent advances in next generation sequencing (NGS) technologies have enabled genome-wide analysis of single cells. Genomic analysis of single cells is being performed in diverse areas such as cancer, prenatal genetic diagnoses for testing success of pregnancies, microbiology, neural diseases, and immunology [8–10]. The purpose of genomic analysis of single cells and the areas in which single cell analysis is being performed is detailed elsewhere [11]. Single cell genomic analysis is used to assess mutations involved in the transformation of normal cells into cancerous cells. For instance, to assess the mutations leading to the transformation of hematopoietic stem cells (HSC) to acute myelogenous leukemia (AML) [12]. The authors investigated the possibility of serial acquisition of mutations in HSC through exome sequencing of sorted single HSC which could act as pre-leukemic HSC supposedly contributing to a reservoir of mutated cells that should be therapeutically targeted in order to successfully treat AML [12]. Genomic variations in somatic cells are known to accumulate during development and increase with aging. Genomic variations in somatic cells are implicated in neurodegenerative diseases such as Alzheimer’s disease. Recent advancement in single cell techniques enables the understanding of somatic mutations that occur in single genomes of the cells of neural tissue leading to better understanding of neurodegenerative disorders [9, 10]. Additionally, single cell analysis can be helpful in better understanding of tumor heterogeneity which is commonly observed in several cancers including prostate cancer [1]. Single CTCs were isolated from prostate cancer patients through MagSweeper technology and mRNA sequencing was performed [13]. Analysis of single cells is also being conducted in the area of diabetes, where in a study the authors performed a novel real-time PCR assay in single cells isolated from pancreas in order to identify the mechanisms leading to progression of type 1 diabetes [14]. Analysis of single cells from human sperm cells and embryos is performed to identify presence of genetic diseases [11, 15]. Sophisticated techniques such as microarray expression assay, RNA sequencing, whole transcriptomic analysis (WTA) and whole genomic analysis (WGA) of single cells are now well developed and are being used to study the genomic and transcriptomic variability in single cells from different cell types [7, 16, 17]. Surani et al. were the first group to report a study based upon WTA of single mouse blastomeres and oocytes [6]. As such, the ability to measure gene expression in single cells can be helpful in understanding the mechanisms leading to disease progression and to develop better therapeutic regimen not only in cancer but also for many other diseases.

In the current study, single cells were isolated based on the side population phenotype from human prostate clinical specimens and a prostate cancer cell line to identify expression of specified genes by reverse transcription PCR (RT-PCR). The side population assay is based on the ATP binding cassette transporter G2 (ABCG2) activity [18]. Previous studies from our and other laboratories have identified side population cells from various prostate cell lines and human prostate clinical specimens [19–23]. We have shown that side population cells isolated from fresh human prostate clinical specimen enrich for prostate stem cells [20]. For the current study, we have isolated single side population and non-side population cells and studied their gene expression using one-step RT-PCR. RT-PCR is one of the most basic and cost effective molecular techniques, thus providing a platform for more sophisticated and sensitive analysis. Although advanced techniques such as microfluidics based system are available to isolate single cells and techniques such as microarray expression assay, RNA sequencing, WTA and WGA of single cells are well developed to study gene expression in single cells, the primary goal of the current study is to provide a technique that could be commonly used, to assess gene expression of single cells, by the scientific community who do not have the resources to perform microfluidic sorting.

## Materials and Methods

### Cell culture

Briefly, the CWR-R1 prostate cancer cells were grown in Ritcher’s improved MEM (Invitrogen, Carlsbad, CA) supplemented with: FBS (2% v/v) (Invitrogen, Carlsbad, CA); Linoleic acid (0.0018 mg/ml) (Sigma Aldrich, St. Louis, MO); ITS (0.2X) (Sigma Aldrich, St. Louis, MO); epidermal growth factor (0.1 μg/ml) (BD Biosciences, San Jose, CA); and penicillin/streptomycin (1% v/v) (Invitrogen, Carlsbad, CA). The cells were grown in an incubator at 37°C at an atmosphere of 5% CO_2_. The cells were grown to 90% confluence before harvested by incubation with trypsin-EDTA for future experimental procedures.

### Human prostate specimen digestion and isolation of epithelial cells

Human prostate digestion and isolation of epithelial cells were performed as described previously [24]. Briefly, fresh human prostate tissue, harvested from radical prostatectomy surgery stored until digestion in static preservation solution (SPS-1™) (Organ Recovery Systems, Buffalo, NY) at 4°C was obtained from the Pathology Resource Network at RPCI. Enzymatic tissue digestion with a mixture of: dispase (2.4 U/ml) (Invitrogen, Carlsbad, CA); DNase (0.01% w/v) (Sigma Aldrich, St. Louis, MO); and collagenase (2.8% w/v) (Sigma Aldrich, St. Louis, MO) followed by single cell suspension in Hanks buffered salt solution (HBSS) (Invitrogen, Carlsbad, CA) supplemented with 5% FBS was prepared as described previously [24].

### Side population assay

**Figure 1:**
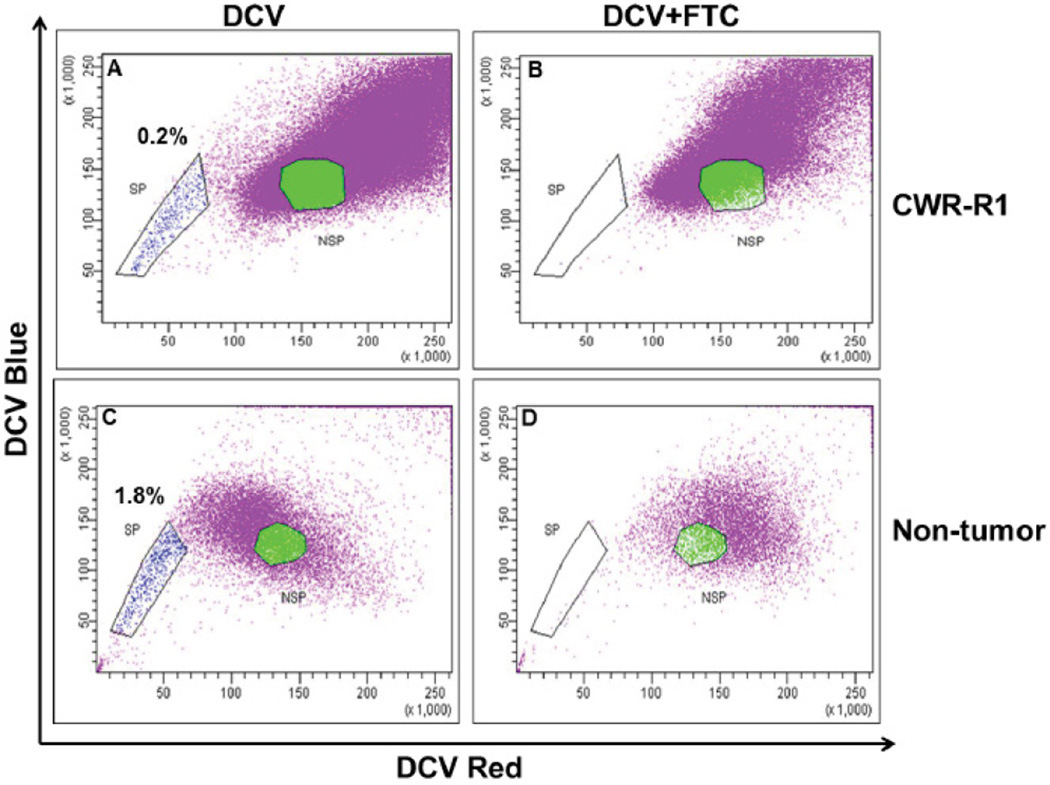
FACS isolation of side population in the CWR-R1 prostate cancer cell line and human non-tumor prostate clinical specimens based upon DCV efflux The side population is gated based upon the ABCG2-mediated efflux of DCV (**A** and **C**). The efflux of DCV is inhibited in the presence of Fumitremorgin C (FTC), a specific inhibitor of ABCG2 (**B** and **D**).

The side population assay was performed as described previously [24]. Briefly, CWR-R1 prostate cancer cells and epithelial cells isolated from freshly digested human prostate specimen were stained with dye cycle violet (DCV) (Invitrogen, Carlsbad, CA) in order to isolate single side population and non-side population cells according to a protocol modified from Telford et al. [25] and as previously described [24]. The assay is based upon the principle of ABCG2 functional activity where the cells with functionally active ABCG2 transporter efflux DCV and fall in the lower fluorescent intensity region of a flow cytograph. The percentage of side population cells obtained from the CWR-R1 prostate cancer cell line and human prostate clinical specimen is shown in Figure 1.

### Isolation of single cells using FACS

A BD FACS Aria II flow cytometer was used to isolate single side population and non-side population cells from the CWR-R1 prostate cancer cell line and epithelial cells isolated from human prostate clinical specimen. Single side- and non-side population cells were collected into 96-well PCR plates containing 5 μl nuclease-free water. Before the collection of single cells, the flow cytometer was calibrated using beads to ensure proper placement of the single cell into each well of the 96-well plate. Settings ensured that the droplet carrying the single cell fell in the center of the well. A sufficient number of cells (a minimum of 5 × 10^4^ cells per sample) should be planned to be stained with the specified marker of detection as several cells are utilized to determine the parameters for the side population assay. A representative figure of the sort layout showing single side- and non-side population cells sorted into a 96-well PCR plate is shown in Supplementary Figure S1. Cells are on ice at all times in order to ensure proper viability of the collected cells. Once cells are collected, the 96-well plate should be sealed with a micro-adhesive film (Invitrogen, Carlsbad, CA) and kept on ice before further processing. Care should be taken not to move the 96-well plate with cells so as to avoid possible disruption of the cells.

Five to ten cells were sorted into three wells of 96-well plate for each experiment to ensure proper calibration of FACS in each run and to have a control amount of RNA to detect expression of genes of interest (data not shown).

### Cell lysis

Single cells were collected in a droplet of flow sheath fluid in a few nanoliters, so there was no significant change in volume of nuclease free water. The cells were immediately heated in a PCR thermocycler machine to 95°C for 5 min to ensure lysis of the cells. The lysed cells were either immediately used for RT-PCR or were stored at −80°C, for a maximum of 14 days, before used for detection of target gene expression using one-step RT-PCR.

### One-step reverse transcription PCR

A major advantage with performing one-step RT-PCR was to eliminate the need for removal of the cDNA from the well of 96-well plate. RT-PCR is a commonly used technique without the need for expensive equipment to detect gene expression. Superscript III one-step reverse transcription kit with Taq polymerase (Invitrogen, Carlsbad, CA) was used in all our experiments. One-step RT-PCR was performed according to the manufacturer’s instructions. Gene-specific primers were used for the one-step RT-PCR reaction (Table 1). Each well contained a single cell in a 10 μl reaction. In order to avoid unnecessary binding of primers to genomic DNA, primers were designed to span introns (Integrated DNA Technologies, Coralville, IA). Expression of ABCG2, ALDH1A1, Oct-4, AR, GAPDH, and actin genes were detected in single side- and non-side population cells sorted from the CWR-R1 prostate cancer cell line and epithelial cells isolated from freshly digested human prostate clinical specimen. One-step RT-PCR was performed according to the manufacturer’s instructions with the following PCR reaction conditions: 45 cycles consisting of (95°C for 30 sec, 60°C for 75 sec, and 72°C for 75 sec). The PCR products were electrophoretically size-fractionated on a 2% agarose gel and the bands were visualized using ethidium bromide. The estimated sizes of the PCR products are listed in Table 1. No RNA in a reaction was used in each experiment as a negative control to validate the conditions of RT-PCR. PCR product sequences were confirmed using capillary gel electrophoresis (data not shown).

### Quantitation of RT-PCR results

**Figure 2:**
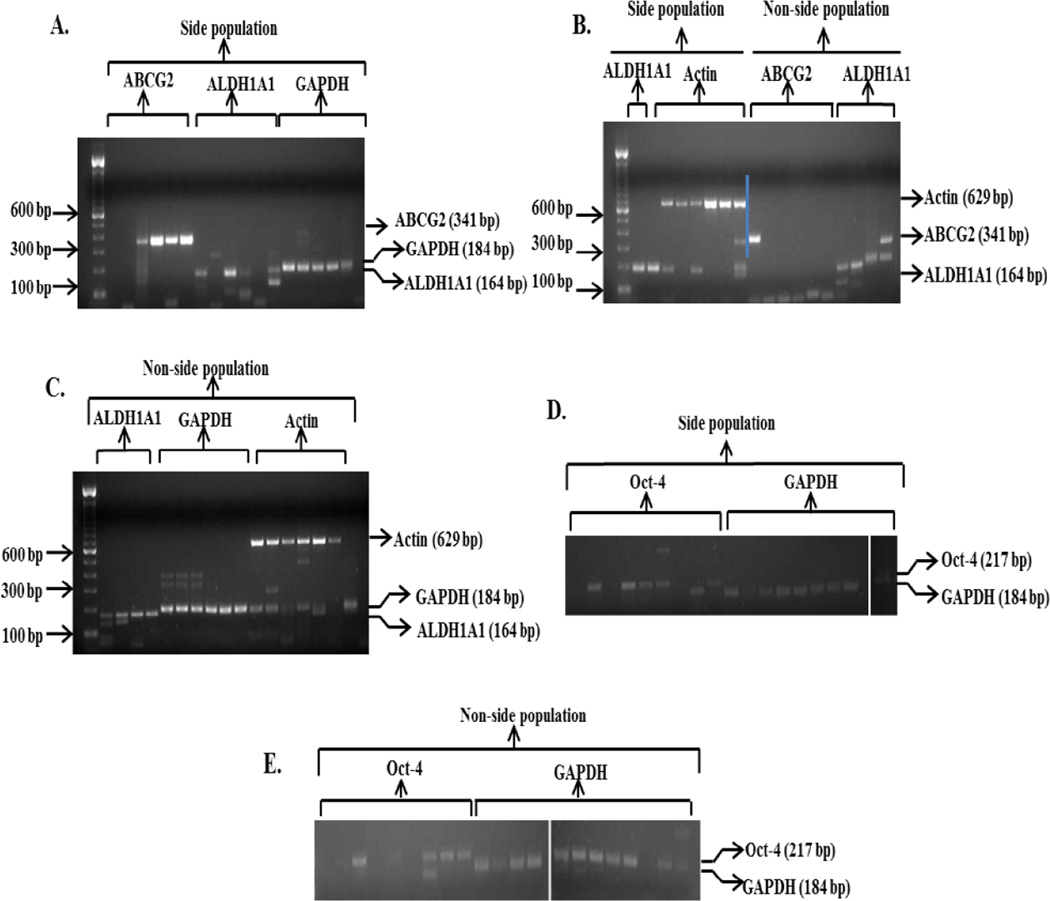
Expression of ABCG2, ALDH1A1, and Oct-4 genes in single side population and non-side population cells isolated from the CWR-R1 prostate cancer cell line Representative data of samples run on 2% agarose gel, Table 3 showing expression of ABCG2, ALDH1A1, Oct-4, GAPDH and actin genes in single side population (**A, B and D**) and single non-side population cells (**B, C and E**) isolated from the CWR-R1 prostate cancer cell line. (**B**) The left side of blue line represents gene expression in side population cells and the right side represents gene expression in non-side population cells.

Intensity of target gene RT-PCR product represented in Figure 2 was quantitated using ImageJ (1.47 v) software [26]. The band areas are equivalent to pixel numbers and represent relative band intensity of the target RT-PCR product. The band areas reported by ImageJ software are relative to the total band size and density of the bands that have been selected.

### Statistical analysis

The success rate in the side and non-side population samples was quantified as the binomial proportion among the observed cells. The 95% confidence interval describes a plausible range for the true success rate that is supported by the data. Confidence intervals were estimated using Jeffrey’s Method, which has favorable properties in this setting [27]. Fisher’s Exact Test quantified evidence against the null hypothesis of no difference in the side- and non-side population success rates. Results from this exploratory study are considered hypothesis generating. P values less than 0.05 were considered statistically significant, without adjustments for multiple testing. All data analyses were generated using SAS/STAT software, Version 9.4. Copyright 2012, SAS Institute Inc. SAS is a registered trademark of SAS Institute Inc., Cary, NC, USA.

## Results

### Optimization of single cell isolation using fluorescence activated cell sorting (FACS) and detection of gene expression using one-step RT-PCR

The goal of the current study is to assess gene expression in single cells using a commonly used technique. RT-PCR has been used in the study as we intended to determine gene expression in single cells using standard equipment and techniques. The results obtained using RT-PCR is expected to be reproducible using quantitative RT-PCR, which is more sensitive. FACS was used for collecting single cells with a specific phenotype. Positioning the cell droplet in the center of the well requires that the FACS instrument is well calibrated, and is critical for proper cell lysis and analysis of cellular content. A reliable cell sorting facility and personnel is required to calibrate the FACS instrument in order to reproducibly sort live single cells based upon markers of interest. If the cell droplet hits the side of the well, the liquid evaporates immediately leading to lysis of the cell, and degradation of RNA. RNA quality and quantity is important to prevent false negative reads of target gene expression. Proper cell lysis is a critical step for isolating quality RNA. The addition of 5 μl of nuclease free water in each well, prior to single cell isolation, worked well in our studies to prevent cell lysis due to water evaporation. Cells were lysed by heating the 96-well plate containing single cell in each well to 95°C for 5 minutes and resulted in a loss of ~1–2 μl nuclease-free water, any time greater than 5 minutes caused evaporation of the nuclease free water.

### ABCG2 and Oct-4 genes are expressed in single side population and non-side population cells isolated from the CWR-R1 prostate cancer cell line

As a proof of concept study, we chose to detect expression of a limited number of genes in single cells. This study examines the genomic heterogeneity in single cells isolated based upon the side population phenotype. We analyzed single side- and non-side population cells in order to compare gene expression data previously demonstrating bulk side population cells have elevated levels of ABCG2 gene expression [19, 20, 28].

**Figure 3:**
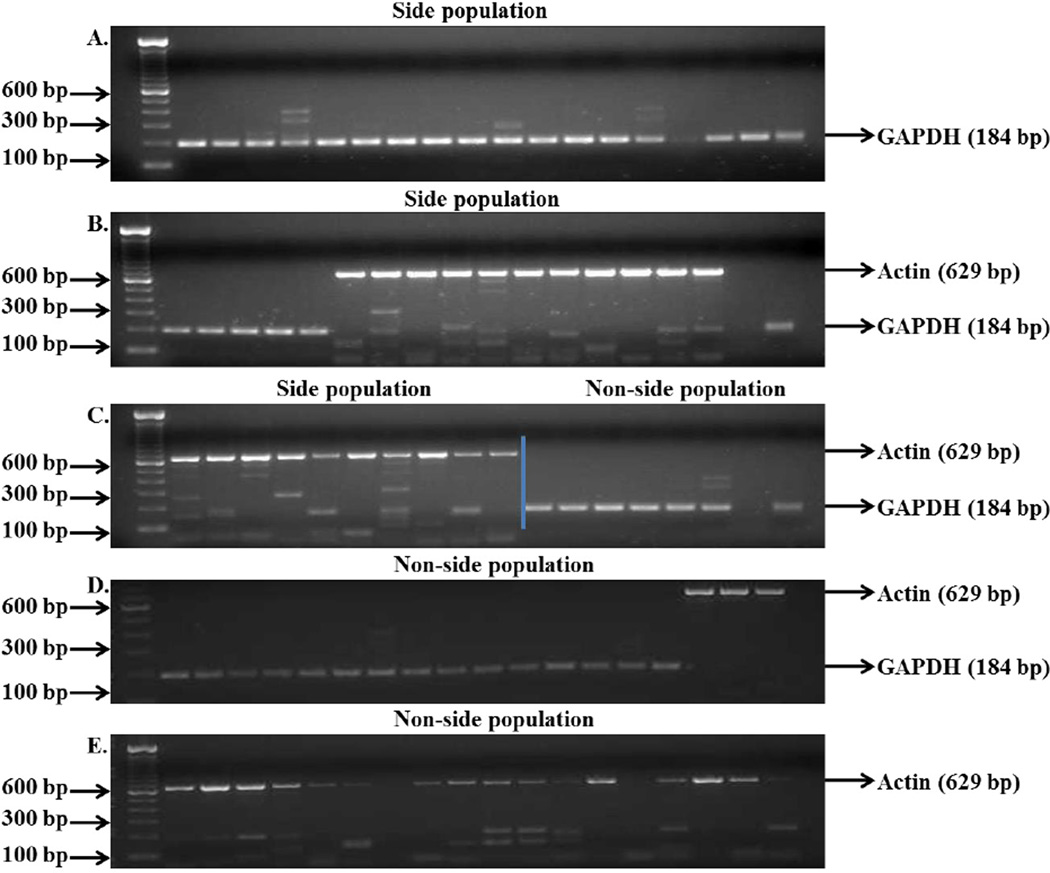
Expression of GAPDH and actin genes in single side population and single non-side population cells isolated from the CWR-R1 prostate cancer cell line Representative data of samples run on 2% agarose gel showing expression of GAPDH and actin genes in single side population (A–C) and single non-side population cells (C–E) isolated from the CWR-R1 prostate cancer cell line. (C) The left side of blue line represents gene expression in side population cells and the right side represents gene expression in non-side population cells.

ABCG2 gene expression was analyzed since side population and non-side population cells are isolated based upon the presence or absence, respectively, of functionally active ABC transporters. ALDH1A1 and Oct-4 gene expression was analyzed because ALDH1A1 is now being widely studied as a prostate cancer stem cell marker [29–31] and Oct-4 is an embryonic stem cell marker [32–34]. Representative data showing the gating strategy of side population obtained from the CWR-R1 prostate cancer cell line is shown in Figure 1A and 1B. Expression of housekeeping genes, GAPDH and Actin, was initially examined in single side- and non-side population cells isolated from the CWR-R1 prostate cancer cell line (Table 2) to optimize the conditions for one-step RT-PCR and also to test the reproducibility and accuracy of the entire procedure from cell collection, cell lysis, and gene expression detection. RNA was isolated from bulk CWR-R1 cells sorted based on the side population or non-side population phenotype and 1 ng to 10 ng was included in all RT-PCR reactions as a positive control. Wells with no RNA were used as a negative control in order to validate conditions used for RT-PCR. A high percentage, 90.5% to 100%, sorted cells from the CWR-R1 prostate cancer cell line demonstrated expression of GAPDH and actin genes in our preliminary experiments (Table 2 and Figure 3). Analysis of the expression of ABCG2, ALDH1A1, and Oct-4 genes in single side- and non-side population cells isolated from the CWR-R1 prostate cancer cell line was performed. Representative data of gene expression in single side- and non-side population cells isolated from CWR-R1 prostate cancer cells is shown in Figure 2.

**Figure 4:**
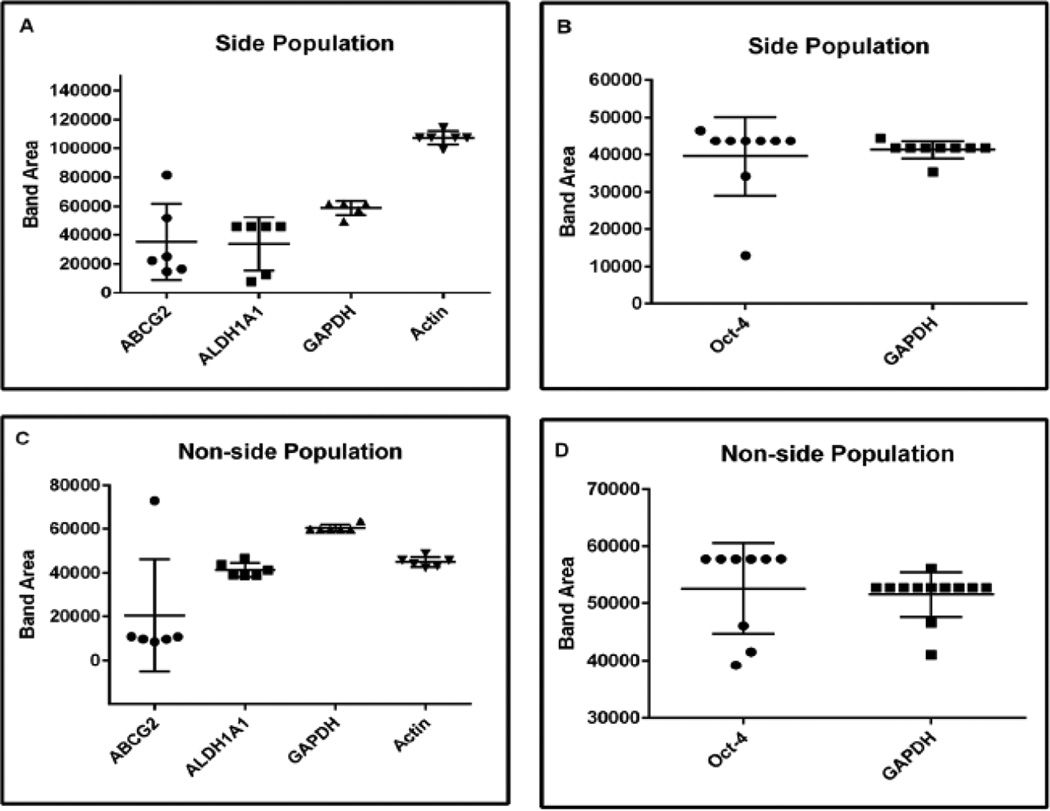
Quantitation of RT-PCR results Intensity of target gene RT-PCR product represented in Figure 2 was quantitated using Image J (1.47 v) software. Each dot in the panels A–D represents relative band intensity of the target/housekeeping gene RT-PCR product tested in single side- or non-side population cell isolated from the CWR-R1 prostate cancer cell line. For example in Figure 4A and B, each dot represents an individual relative band intensity of ABCG2, ALDH1A1, Oct-4, GAPDH or actin RT-PCR product in single side population cells. In Figure 4C and D, each dot represents an individual relative band intensity of ABCG2, ALDH1A1, and Oct-4, GAPDH or actin RT-PCR product in single non-side population cells.

Results from the current study demonstrate that though there were no statistically significant differences in expression, a higher percentage of single side population cells express the ABCG2 gene compared to single non-side population cells. ABCG2 gene expression is detected in 67% of single side population cells as compared to 17% of single non-side population cells expressing ABCG2 gene (p=0.24) (Table 3, Experiment 1). While ALDH1A1 gene expression is seen in 100% single non-side population cells, 67% of single side population cells expressed ALDH1A1 gene (p=0.45) (Table 3, Experiment 1). In a separate experiment, the percentage of single side- and non-side population cells expressing Oct-4 gene was determined. Oct-4 gene expression was detected in 56% of single side population cells and in 45% of single non-side population cells (p=1.00) (Table 3, Experiment 2). Relative band intensities of ABCG2, ALDH1A1, and Oct-4 RT-PCR products were quantitated using ImageJ software [26] and the results were represented in Figure 4.

### Oct-4 gene is expressed in a lower percentage of single side population cells isolated from human prostate specimen compared to single non-side population cells

To analyze markers that are not expected to be expressed in the same cell, Oct-4 and AR gene expression was analyzed in single cells isolated from human prostate tissue. We examined the expression of AR and Oct-4 genes in single side- and non-side population cells isolated from two non-tumor human prostate clinical specimens obtained from radical prostatectomy surgeries. Representative data showing the gating strategy of side population obtained from human prostate clinical specimens is shown in Figure 1C and 1D. GAPDH is expressed in 100% single side- and non-side population cells (Table 4). While AR gene expression is observed in 100% single side- and non-side population cells, Oct-4 gene expression is observed in 23% of single side population cells and 78% of single non-side population cells expressed Oct-4 gene (p<0.01) (Table 4). Thus, results using single cells isolated from human prostate clinical specimens showed that AR is expressed in all cells analyzed (p value not available). There is a statistically significant difference in the expression of Oct-4 gene between the two sorted populations (p<0.01).

Results from the current study demonstrated that ABCG2, ALDH1A1, and Oct-4 genes are expressed heterogeneously in single cells isolated from the CWR-R1 prostate cancer cell line (Table 3), a difference in gene expression would be hard to detect when analyzing bulk cells. Oct-4 gene expression was detected in a low percentage of single side population cells as compared to single non-side population cells isolated from human prostate clinical specimen (Table 4). Finally, results demonstrate that the cells isolated based on the side- and non-side population phenotype from CWR-R1 prostate cancer cells have a heterogeneous expression of ABCG2, Oct-4, and ALDH1A1. Cells isolated based on the side- and non-side population phenotype from human prostate clinical specimens have homogenous expression of AR and heterogeneous expression of Oct-4. Thus, the results demonstrate that single cell isolation using FACS followed by one-step RT-PCR can be used as an effective tool to determine gene expression in single cells.

## Discussion

Single cell analysis can determine the changes in gene expression associated with clonal development and tumor heterogeneity which are the likely underlying reasons for therapy resistance and cancer recurrence [1, 3]. Single cell analysis is also helpful in delineating the cellular hierarchy within a tumor or an organ [4, 5, 7]. Bulk population measurements often can lead to misinterpretation of gene expression analysis [16, 17]. Though there are several studies which have been successful in utilizing new technology and performing different types of analyses such as WGA, whole exome analysis, and mRNA sequencing on single cells, evidently, there are limitations to conduct such studies such as: large amount of clinical specimen is needed to isolate the required number of cells; expensive equipment and supplies; technical expertise; time constraints; reproducibility; and reliability. Thus, in our current study we have utilized a relatively low cost, simple, and reproducible technique to analyze single cell gene expression. We have analyzed expression of ABCG2, ALDH1A1, AR, and Oct-4 genes in single prostate epithelial cells.

We have analyzed gene expression in single cells isolated from the CWR-R1 prostate cancer cell line and two non-tumor human prostate clinical specimens using RT-PCR. RT-PCR has been employed as we intended to use commonly available equipment and standard techniques to detect gene expression in single cells. Since single cells have low amounts of starting RNA for downstream gene expression analysis, isolating live and intact single cells with precision was warranted. FACS is an advanced technique best suitable to isolate single cells of interest with precision in less time compared to other single cell isolation techniques such as microfluidics. An advantage in using FACS for single cell isolation is that a higher number of live and intact cells can be collected into a 96-well plate within a few minutes once settings are established. Live cells can be collected and dead cells eliminated by staining cells with 7-aminoactinomycin D (7-AAD). A disadvantage associated with utilizing single cells isolated from patient samples is: variations of gene expression due to cell cycle [35]. Through our recent studies we have observed that time between devascularization and procurement does not have an effect on tissue quality, as defined by cell number and RNA integrity number (RIN) values [36].

Results from the current study showed that there is no statistically significant difference in ABCG2 or ALDH1A1 gene expression in single side population cells isolated from the CWR-R1 prostate cancer cell line compared to single non-side population cells (Table 3). Previous studies from our laboratory have demonstrated that the ABCG2 gene is highly expressed (five-fold increase) in bulk population of CWR-R1 side population cells compared to non-side population cells [28]. Whereas, ALDH1A1 gene is highly expressed (thirty-fold increase) in bulk population of CWR-R1 non-side population cells compared to that observed in side population cells [28]. We do not know if both ABCG2 and ALDH1A1 genes are expressed in a given single side population or single non-side population cell for which multiplexing is recommended. Furthermore, the 33% single side population cells without ABCG2 gene expression have the capability to efflux DCV dye and fall as a “side” population in a flow cytograph. This observation suggests two different possibilities: (i) though ABCG2 is known to be a primary molecular determinant of side population [37], there could be transporters other than ABCG2 such as *Mdr-1* highly expressed in side population cells [38] that can contribute to the “side” population; or (ii) though the single side population cells possess functionally active ABCG2 transporter as evidenced by their ability to efflux DCV, the ABCG2 gene is not expressed in 100% side population cells suggesting that the presence of a functionally active protein does not have to correlate with the gene expression level [39, 40]. There is a lower percentage (17%) of single non-side population cells expressing ABCG2 gene and 100% single non-side population cells expressed ALDH1A1 gene suggesting differential gene expression in non-side population cells (Table 3). Such heterogeneity in gene expression in side- and non-side population cells is easily detected with single cell analysis. While some variability was noted in relative band intensities of ABCG2, ALDH1A1, and Oct-4 RT-PCR products, there was little variability noted in the relative band intensities of GAPDH and actin RT-PCR products in single side population and single non-side population cells isolated from the CWR-R1 prostate cancer cell line (Figure 4).

Oct-4 gene expression was detected in a low percentage of single side population cells as compared to single non-side population cells isolated from human prostate clinical specimen (Table 4), while no difference is observed between percentages of single side- and non-side population cells expressing the AR gene.

## Conclusions

In the current study, we demonstrated a technique involving a series of steps which enabled the isolation of single cells to identify gene expression in a single side population or a single non-side population cell. FACS combined with RT-PCR provides a straight-forward procedure to isolate single cells and detect gene expression. Though highly context dependent, variability of the response to external stimulus by single cells in a given population of cells, quantitative measurements of genes expressed in single cells because of the external stimulus may become important. In such instances, we recommend the performance of real time PCR, a technique with high sensitivity, rather than RT-PCR in order to understand response of single cells to the external stimulus. Nonetheless, RT-PCR would be a good technique to follow in the context of identifying the presence or absence of gene expression in single cells and when the consequence of the gene expression i.e., changes in gene expression levels or the result of a change in gene expression level is not the final intended measurement. Although still in the developmental stages, single cell analysis has the potential to aid in advancing our understanding of disease. Thus, the measurement of different parameters of single cells such as genome, epigenome, proteome, and metabolome would enable to study the mechanisms leading to transformation of an otherwise normal organ. Therefore, the purpose of our study is to provide a straight forward technique which enables identification of gene expression in single cells.

## Supplementary Material

01

## Figures and Tables

**Table 1:** Primer sequences and the estimated sizes of the PCR products.

Name	Sequence (5’-3’)	Product Length (bp)
ABCG2-F	AGCAGGATAAGCCACTCATAGA	341
ABCG2-R	GTTGGTCGTCAGGAAGAAGAG	
ALDH1A1-F	TTACCTGTCCTACTCACCGATT	164
ALDH1A1-R	GCCTTGTCAACATCCTCCTTAT	
AR-F	TACCAGCTCACCAAGCTCCT	195
AR-R	GCTTCACTGGGTGTGGAAAT	
Oct-4-F	CCTGTCTCCGTCACCACTC	217
Oct-4-R	CACCTTCCCTCCAACCAGTT	
GAPDH-F	GAACATCATCCCTGCCTCTACT	184
GAPDH-R	CGCCTGCTTCACCACCTT	
Actin-F	ACTCTTCCAGCCTTCCTTCC	629
Actin-R	CTTCCTGTAACAACGCATCTCA	

**Table 2:** Percentage of single side- and non-side population cells isolated from the CWR-R1 prostate cancer cell line expressing housekeeping genes, GAPDH and Actin.

CWR-R1 prostate cancer cells	Gene (primer concentration)	SP		NSP		p-value
		*% Gene expression	** 95% CI	* %Gene expression	** 95% CI	
Experiment 1	GAPDH (0.1 µM)	9/9 (100)	76.2 to 100	9/9 (100)	76.2 to 100	na
Experiment 2	GAPDH (0.1 µM)	21/21 (100)	88.9 to 100	21/21 (100)	88.9 to 100	na
	Actin (0.1 µM)	21/21 (100)	88.9 to 100	19/21 (90.5)	72.8 to 98	0.49

P values were determined by Fisher’s Exact test.

* % gene expression represented in parentheses is the total number of samples expressing gene/total samples run per gene.

** The 95% confidence interval (CI) describes a plausible range for the true success rate that is supported by the data. 95% confidence intervals for the successful proportion were determined by Jeffrey’s method.

**Table 3:** Percentage gene expression in single side- and non-side population cells isolated from the CWR-R1 prostate cancer cell line.

CWR-R1 prostate cancer cells	Gene (primer concentration)	SP		NSP		
		*%Gene expression	**95% CI	*%Gene expression	**95% CI	p value
Experiment 1	ABCG2 (0.4 µM)	4/6 (66.7)	28.6 to 92.3	1/6 (16.7)	1.9 to 55.8	0.24
	ALDH1A1 (1 µM)	4/6 (66.7)	28.6 to 92.3	6/6 (100)	67.0 to 100	0.45
	GAPDH (0.1 µM)	6/6 (100)	67 to 100	6/6 (100)	67 to 100	na
	Actin (0.1 µM)	6/6 (100)	67 to 100	6/6 (100)	67 to 100	na
Experiment 2	GAPDH (0.1 µM)	9/9 (100)	76.2 to 100	11/12 (92)	67.2 to 99.1	1.00
	Oct-4 (0.8 µM)	5/9 (56)	25.4 to 82.7	4/9 (45)	17.3 to 74.6	1.00

P values were determined by Fisher’s Exact test.

**Table 4:** Percentage gene expression in single side- and non-side population cells isolated from human prostate clinical specimens.

Specimen	Gene (primer concentration)	SP		NSP		p value
		*% Gene expression	**95% CI	*% Gene expression	**95% CI	
Non-tumor prostate	GAPDH (0.8 µM)	12/12 (100)	81.5 to 100	12/12 (100)	81.5 to 100	na
	AR (0.2 µM)	8/8 (100)	73.8 to 100	8/8 (100)	73.8 to 100	na
	Oct-4 (0.8 µM)	4/18 (22.2)	8 to 44.6	14/18 (77.7)	55.4 to 92	<0.01

P values were determined by Fisher’s Exact test.

## Abbreviations

DCV: Dye Cycle Violet
ABCG2: ATP Binding Cassette Transporter G2
ALDH1A1: Aldehyde Dehydrogenase 1A1
RT-PCR: Reverse Transcription Polymerase Chain Reaction
AR: Androgen Receptor
CTC: Circulating Tumor Cell
NGS: Next Generation Sequencing
WTA: Whole Transcriptomic Analysis
WGA: Whole Genome Analysis
RIN: RNA Integrity Number
FACS: Fluorescence Activated Cell Sorting
7-AAD: 7-Aminoactinomycin D
RPCI: Roswell Park Cancer Institute
IRB: Institutional Review Board
SPS-1™: Static Preservative Solution-1
HBSS: Hanks Buffered Salt Solution
GAPDH: Glyceraldehyde-3-Phosphate Dehydrogenase
SP: Side Population
NSP: Non-Side Population
