# Supplementary material for: Gene Expression in Single Cells Isolated from the CWR-R1 Prostate Cancer Cell Line and Human Prostate Tissue Based on the Side Population Phenotype
Source: Single Cell Biol. Author manuscript; Available in PMC 2016 Oct 24. (PMC5076885; doi:10.4172/2168-9431.1000150)
Supplement: 01 [file NIHMS824443-supplement-01.pdf]

Supplemental Figure S1: Sort layout showing the number of side population and non-side population cells collected into each well of a 96-well PCR plate: Single side population or single non-side population cell was collected into each well of a 96-well PCR plate. In each plate some wells (wells D9-D12 and H9-H12 in the plate below) were left empty in order to load positive control RNA or no RNA control in order to validate conditions for RT-PCR. The sort layout represents single side population and non-side population cells isolated from human prostate clinical specimen. SP – Side population; Non – Non-side population

Sort Layout

	1	2	3	4	5	6	7	8	9	10	11	12
A	SP:1/1	SP:1/1	SP:1/1	SP:1/1	SP:1/1	SP:1/1	SP:1/1	SP:1/1	SP:1/1	SP:1/1	SP:1/1	SP:1/1
B	SP:1/1	SP:1/1	SP:1/1	SP:1/1	SP:1/1	SP:1/1	SP:1/1	SP:1/1	SP:1/1	SP:1/1	SP:1/1	SP:1/1
C	SP:1/1	SP:1/1	SP:1/1	SP:1/1	SP:1/1	SP:1/1	SP:1/1	SP:1/1	SP:1/1	SP:1/1	SP:1/1	SP:1/1
D	SP:1/1	SP:1/1	SP:1/1	SP:1/1	SP:1/1	SP:1/1	SP:1/1	SP:1/1				
E	non:1/1	non:1/1	non:1/1	non:1/1	non:1/1	non:1/1	non:1/1	non:1/1	non:1/1	non:1/1	non:1/1	non:1/1
F	non:1/1	non:1/1	non:1/1	non:1/1	non:1/1	non:1/1	non:1/1	non:1/1	non:1/1	non:1/1	non:1/1	non:1/1
G	non:1/1	non:1/1	non:1/1	non:1/1	non:1/1	non:1/1	non:1/1	non:1/1	non:1/1	non:1/1	non:1/1	non:1/1
H	non:1/1	non:1/1	non:1/1	non:1/1	non:1/1	non:1/1	non:1/1	non:1/1				
